# Cleidocranial dysplasia with growth hormone deficiency: a case report

**DOI:** 10.1186/s12887-020-1914-8

**Published:** 2020-01-16

**Authors:** Nozomi Takaki, Jun Mori, Satoshi Matsuo, Toshio Osamura, Toshimi Michigami

**Affiliations:** 1 Department of Pediatrics, Kyoto Prefectural University of Medicine, 465 Kajii-cho Kawaramachi-Hirokoji, Kamigyo-ku, Kyoto, Japan; 2 Matsuo Kids Clinic, Kyoto, Japan; 3 Department of Pediatrics, Japanese Red Cross Kyoto Daini Hospital, Kyoto, Japan; 4 Department of Bone and Mineral Research, Research Institute, Osaka Women’s Children’s Hospital, Osaka Prefectural Hospital Organization, Izumi, Japan

**Keywords:** Cleidocranial dysplasia, Short stature, *RUNX2*, Growth hormone deficiency

## Abstract

**Background:**

Cleidocranial dysplasia (CCD) is a rare skeletal disorder with autosomal dominant inheritance that is characterized by hypoplastic clavicles, delayed closure of the cranial sutures, dental abnormalities, and short stature, among other features. The responsible gene for CCD is *RUNX2* located on the short arm of chromosome 6p21. In general, there are intrafamilial variations in height among CCD patients. Few studies have reported data on recombinant human growth hormone (rhGH) treatment for patients with CCD; thus, it remains to be elucidated whether rhGH treatment can improve short stature. Here, we report a case of a 6-year-old girl with CCD who has growth hormone deficiency (GHD) and a novel mutation of *RUNX2*.

**Case presentation:**

At 5 years of age, this patient was diagnosed with GHD and rhGH treatment was initiated. Thereafter, she was diagnosed with CCD due to the presence of hypoplastic clavicles and an open fontanelle, which was also observed in her mother and brother. She responded well to rhGH treatment; her height improved from − 3.2 SD to − 2.4 SD after 13 months.

**Conclusion:**

A detailed patient history and physical examination are necessary for the early diagnosis of CCD. Similarly, to ascertain the effect of rhGH treatment, careful evaluation of the patient’s final height post-therapy is needed.

## Background

Cleidocranial dysplasia (CCD) is a rare skeletal disorder with autosomal dominant inheritance, which is characterized by abnormalities in systemic membranous ossification. The characteristic symptoms are hypoplastic clavicles, delayed closure of the fontanelles, delayed eruption of primary and permanent dentition, and short stature. Considerable phenotypic variation has been reported, even within the families [1]. The frequency of CCD is 1 in 1,000,000. Mutations of runt-related transcription factor 2 (*RUNX2*), also known as core-binding factor alpha-1 (*CBFA1*), located on 6p21, can cause CCD [2, 3]. Herein, we report a 6-year-old girl with CCD and accompanying growth hormone deficiency (GHD). She had improved growth velocity after GH treatment.

## Case presentation

The patient was born uneventfully at a gestational age of 40 weeks and 3 days. Her weight, length, and head circumference at birth were 3112 g (− 0.15 SD), 48.5 cm (− 0.89 SD), and 33 cm (− 0.62 SD), respectively. There was no abnormality in screening for inborn errors of metabolism. Her father’s height was 171 cm, while her mother’s was 157 cm. There was no family history of endocrine disorders or metabolic bone diseases. When she was 3 months old, she was referred to our hospital because of a large anterior fontanelle. Her head circumference was within normal range; thus, no further testing was done.

When she was 3 years and 9 months old, she was presented to our hospital with short stature. Her height and weight were 11.5 kg (− 2.24 SD) and 88.3 cm (− 2.69 SD), respectively. Blood test revealed a low level of serum insulin-like growth factor-1 (IGF-1) (24 ng/mL). She had poor growth velocity, with her height SD score decreasing to − 3.2 SD at 5 years and 5 months of age. GH provocation tests were performed to evaluate for GHD. The maximal GH responses to arginine and GHRP-2 were 3.26 ng/mL and 2.97 ng/mL, respectively. Based on these results, she was diagnosed with GHD, and recombinant human GH treatment (0.175 mg/kg/week) was started. Her wrist X-ray showed her supposed bone age at 5 years and 8 months, according to the Japanese TW2-RUS method, was only 3 years and 10 months. Brain MRI showed no morphological abnormalities of the pituitary gland.

**Fig. 1:**
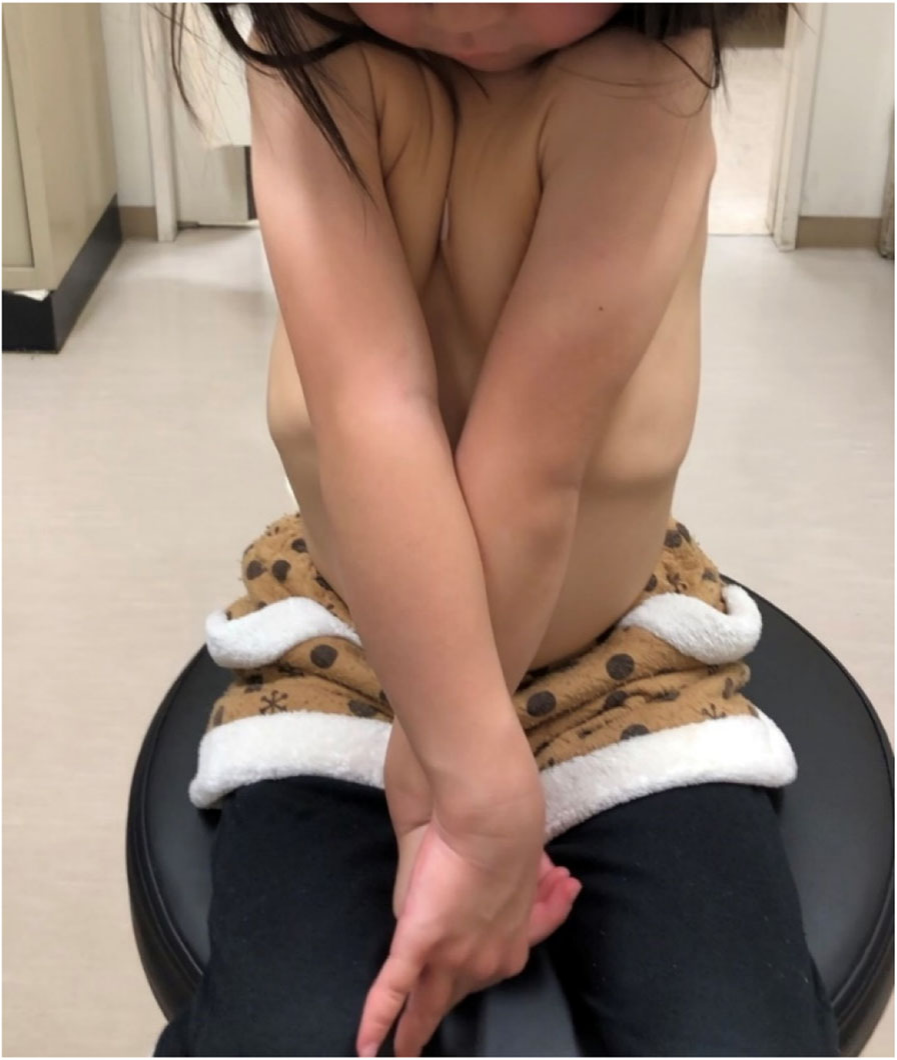
Typical clinical findings in the CCD patient: a prominent forehead, eyelid separation, head larger than the maxillofacial area, and drooping shoulders. Her shoulders are apposed due to the shortened distal portion of her right clavicle

**Fig. 2:**
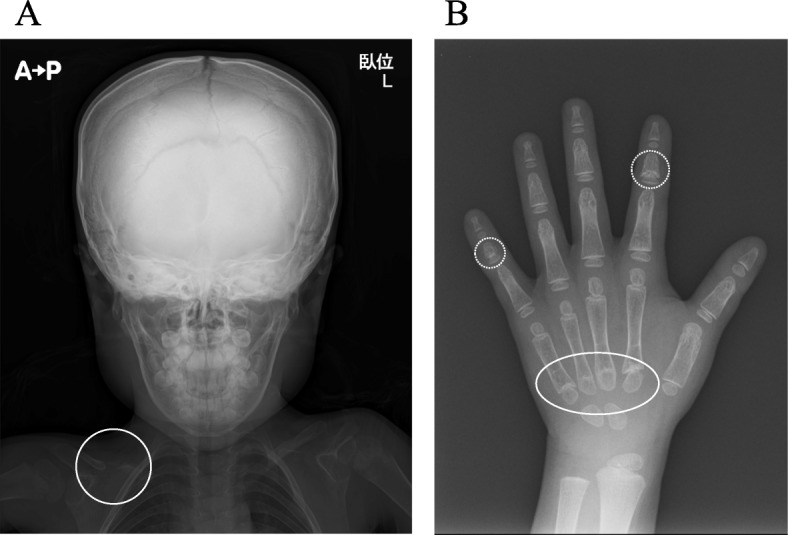
Characteristic findings in the CCD patient. **a** Skull X-ray shows an open anterior fontanelle and hypoplasia of the right clavicle (white circle). **b** Wrist X-ray image shows hypoplasia of the middle phalanges (white ellipse) and pseudoepiphyses of the second and fifth metacarpals (dotted circles)

**Fig. 3:**
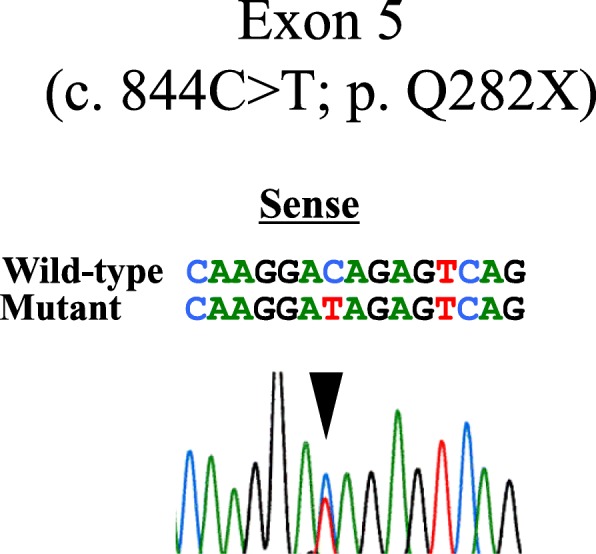
Chromatogram showing the *RUNX2* mutation identified in the present case (c. 844C > T, p. Q282X). The arrowhead shows the mutated nucleotide

When she was 6 years and 2 months old, her mother complained that her 7-month old younger brother also had a large anterior fontanelle. Similarly, the mother had a large anterior fontanelle during her infancy. Thus, we suspected the patient to have a hereditary disease and examined her again. We noted that she had characteristic features of CCD —a large anterior fontanelle, prominent forehead, eyelid separation, head larger than the maxillofacial area, and drooping shoulders. She had apposed shoulders due to a shortened distal portion of her right clavicle (Fig. 1). Her mother and younger brother also had similar characteristic facies. Bone radiographic imaging showed hypoplasia of the right clavicle, open anterior fontanelle, and multiple Wormian bones (Fig. 2a). A hand X-ray showed hypoplasia of the middle phalanges and pseudoepiphyses of the second and fifth metacarpals (Fig. 2b). The *RUNX2* gene was analyzed by Sanger sequencing. Genomic DNA was extracted from peripheral blood leukocytes, and polymerase chain reaction (PCR) was performed to amplify each exon using the primer sets shown in Additional file 1: Table S1. The amplified fragments were then gel-purified and directly sequenced with BigDye® Terminator v1.1 cycle sequencing kit (Applied Biosystems, Foster City, CA, USA). The data were collected on an ABI 3130 Gene Analyzer (Applied Biosystems). The exons, bases, and amino acids were numbered based on a previous report [4]. *RUNX2* gene analysis revealed a heterozygous mutation ‘p. Q282X’ (c. 844C > T) (Fig. 3). The patient’s mother and her younger brother also had the same *RUNX2* gene mutation. She had improved height velocity after initiating GH treatment, as shown by an increase in her height SD score to − 2.4 SD after 13 months of treatment. There were no adverse events associated with rhGH therapy. She is satisfied with the effect of rhGH therapy, as she wants to be tall.

## Discussion and conclusions

CCD is a rare skeletal disorder characterized by hypoplastic clavicles, delayed closure of fontanelles, delayed eruption of primary and permanent dentition, and short stature. It is diagnosed clinically. The prevalence is 1 in 1,000,000. The gene responsible for pathogenesis is *RUNX2* located on the short arm of chromosome 6p21. Genetic analysis reveals a heterozygous mutation of *RUNX2* in almost 70% of patients [5]. *RUNX2* is a transcription factor belonging to the Runx family (Runx1, Runx2, Runx3), and is expressed in osteoblasts and chondrocytes. *RUNX2* is essential for the differentiation of multipotent mesenchymal cells to osteoblasts. It cooperates with Sp7 and canonical Wnt pathway [6]. Bone is formed through two types of ossification: intramembranous or endochondral. For endochondral ossification, *RUNX1* and *RUNX3* can compensate for the *RUNX2* function to some extent, even if the expression of *RUNX2* is halved due to mutation. However, for intramembranous ossification, *RUNX2* is crucial. Thus, open fontanelles and sutures, together with hypoplastic clavicles, are characteristic findings in CCD since the formation of these bones is related to intramembranous ossification [6]. A report in Argentina stated that out of 37 cases studied, 95% had skull abnormalities, 75% had single clavicle alterations, and 100% had bilateral clavicle alterations [7]. The present case also has open fontanelles and hypoplastic clavicles. Interestingly, her mother and brother have open fontanelles, but not hypoplastic clavicles, even though they have the same mutation. This clearly shows that mutations in *RUNX2* could lead to various phenotypic features even in the same family, as previously reported [8].

The present case has a short stature (− 3.2 SD), one of the characteristic features of CCD. The final height of patients with CCD is reported to be − 1.47 SD for men and − 1.89 SD for women [7]. In terms of height, the genotype-phenotype correlation exists in patients with CCD. Patients with mutations in Runt domain have significantly short statures, but patients with intact Runt domain have milder manifestations [1]. The *RUNX2* mutation in the present case is a novel nonsense mutation with intact Runt domain. Her short stature could be attributable to a nonsense mutation. However, her mother with the same mutation has a normal height. This is compatible with a previous paper reporting probable intrafamilial variabilities in height [7]. Her significant short stature could be partially due to GHD. Despite being otherwise healthy, the results of GH provocation tests showed a GHD. The complication of GHD delayed the diagnosis of CCD in the present study. In fact, the early diagnosis of CCD is somewhat difficult because of mild symptoms [7]. A detailed patient history and physical examination are necessary for the early diagnosis of CCD. There are few papers reporting data on rhGH treatment for patients with CCD. The approved therapeutic dose of rhGH to GHD in Japan, 0.175 mg/kg/week, improved the growth velocity in this case [9]. Some papers report the benefit of rhGH treatment in patients with CCD [10], while others refute it [11]. However, the dose used is inconsistent. Thus, it remains unclear whether rhGH treatment can improve the final height [11]. The effect of rhGH might be varying depending on the genotype, as CCD has a wide variability in phenotype. GH produces IGF1, the mediator of protein anabolic and linear growth promoting effect, through Janus activating kinase 2 (JAK2)/ Signal transducer and activator of transcription 5 (STAT5) signaling. STAT5 interacts with *RUNX2* for the differentiation of osteoblast [12]. Thus, if transcriptional activity of *RUNX2* decreases due to gene mutation, GH signaling does not work properly and leads to growth hormone deficiency. Additionally, the effect of rhGH might vary depending on the activity of *RUNX2* based on the genotype. Randomized controlled studies are needed to evaluate the effects of rhGH therapy inpatients with CCD.

In the present study, we have reported a case of CCD with a novel mutation in *RUNX2* and GHD. The family history should be evaluated in detail, and characteristic features of CCD, such as clavicle hypoplasia and open fontanelles, should be checked for when a patient with short stature is encountered. There is a risk of misdiagnosis because of mild symptoms due to variation in phenotypes. Although rhGH treatment was effective in our case, further studies, such as randomized controlled trials, are warranted to reveal the effectiveness and safety of rhGH treatment in CCD patients.

## Supplementary information

**Additional file 1: Table S1.** Primer sets used for the *RUNX2* mutation analysis.

## Data Availability

Informed consent was obtained from the patient and her parents for data and material in this study.

## Abbreviations

CCD: Cleidocranial dysplasia
GHD: Growth hormone deficiency
rhGH: recombinant human growth hormone
*RUNX2*: Runt-related transcription factor 2
