# Supplementary material for: Cleidocranial dysplasia with growth hormone deficiency: a case report
Source: BMC Pediatr. 2020 Jan 16;20:19. doi: 10.1186/s12887-020-1914-8 (PMC6966812; doi:10.1186/s12887-020-1914-8)
Supplement: Supplementary file 1 — Additional file 1: Table S1. Primer sets used for the RUNX2 mutation analysis. [file 12887_2020_1914_MOESM1_ESM.docx]

**Supplemental Table 1.** Primer sets used for the *RUNX2* mutation analysis.

| Exon | Forward primer sequence (5'–3') | Reverse primer sequence (5'–3') |
| --- | --- | --- |
| 0 | ACAGGTTCATATCTTACAGG | GCAGTTTATCAAAGAATCATACC |
| 1 | TGGCTGTTGTGATGCGTATT | TAGCCTCTTACCTTGAAGGCCAC |
| 2 | CAGATGCTTCATTCCTGTCGG | GTGCTGATTTGTATACAGACTAG |
| 3 | TCATTGCCTCCTTAGAGATGC | GGACATGAAAGTGACACTAAC |
| 4 | AATGCTGGCCACCAGATACCG | AATAAGCCGCTTCACAGCTCC |
| 5 | TAAGGCTGCAATGGTTGCTAT | GTCACTGTGAGCATGGATGAG |
| 6 | CTCTGGGAAATACTAATGAGG | AGTGCCATGATGTGCATTTGTAAT |
| 7 | TGTGGCTTGCTGTTCCTTTATG | AGATACCACTGGGCCACTGC |
